# Large Language Models Can Enable Inductive Thematic Analysis of a Social Media Corpus in a Single Prompt: Human Validation Study

**DOI:** 10.2196/59641

**Published:** 2024-08-29

**Authors:** Michael S Deiner, Vlad Honcharov, Jiawei Li, Tim K Mackey, Travis C Porco, Urmimala Sarkar

**Affiliations:** 1 Department of Ophthalmology and Francis I Proctor Foundation University of California San Francisco San Francisco, CA United States; 2 Center for Vulnerable Populations Zuckerberg San Francisco General Hospital Department of Medicine, University of California San Francisco San Francisco, CA United States; 3 Division of General Internal Medicine Zuckerberg San Francisco General Hospital Department of Medicine, University of California San Francisco San Francisco, CA United States; 4 S-3 Research, LLC San Diego, CA United States; 5 Global Health Program, Department of Anthropology University of California San Diego La Jolla, CA United States; 6 Departments of Ophthalmology, Epidemiology and Biostatistics, Global Health Sciences, and Francis I Proctor Foundation University of California San Francisco San Francisco, CA United States

**Keywords:** generative large language model, generative pretrained transformer, GPT, Claude, Twitter, X formerly known as Twitter, social media, inductive content analysis, COVID-19, vaccine hesitancy, infodemiology

## Abstract

**Background:**

Manually analyzing public health–related content from social media provides valuable insights into the beliefs, attitudes, and behaviors of individuals, shedding light on trends and patterns that can inform public understanding, policy decisions, targeted interventions, and communication strategies. Unfortunately, the time and effort needed from well-trained human subject matter experts makes extensive manual social media listening unfeasible. Generative large language models (LLMs) can potentially summarize and interpret large amounts of text, but it is unclear to what extent LLMs can glean subtle health-related meanings in large sets of social media posts and reasonably report health-related themes.

**Objective:**

We aimed to assess the feasibility of using LLMs for topic model selection or inductive thematic analysis of large contents of social media posts by attempting to answer the following question: Can LLMs conduct topic model selection and inductive thematic analysis as effectively as humans did in a prior manual study, or at least reasonably, as judged by subject matter experts?

**Methods:**

We asked the same research question and used the same set of social media content for both the LLM selection of relevant topics and the LLM analysis of themes as was conducted manually in a published study about vaccine rhetoric. We used the results from that study as background for this LLM experiment by comparing the results from the prior manual human analyses with the analyses from 3 LLMs: GPT4-32K, Claude-instant-100K, and Claude-2-100K. We also assessed if multiple LLMs had equivalent ability and assessed the consistency of repeated analysis from each LLM.

**Results:**

The LLMs generally gave high rankings to the topics chosen previously by humans as most relevant. We reject a null hypothesis (*P*<.001, overall comparison) and conclude that these LLMs are more likely to include the human-rated top 5 content areas in their top rankings than would occur by chance. Regarding theme identification, LLMs identified several themes similar to those identified by humans, with very low hallucination rates. Variability occurred between LLMs and between test runs of an individual LLM. Despite not consistently matching the human-generated themes, subject matter experts found themes generated by the LLMs were still reasonable and relevant.

**Conclusions:**

LLMs can effectively and efficiently process large social media–based health-related data sets. LLMs can extract themes from such data that human subject matter experts deem reasonable. However, we were unable to show that the LLMs we tested can replicate the depth of analysis from human subject matter experts by consistently extracting the same themes from the same data. There is vast potential, once better validated, for automated LLM-based real-time social listening for common and rare health conditions, informing public health understanding of the public’s interests and concerns and determining the public’s ideas to address them.

## Introduction

### Public Health Insights From Social Media

Social media platforms can shed light on public health trends and patterns to inform targeted interventions and communication strategies [1]. The potential to leverage social media to better understand public sentiment about vaccines, which play a crucial role in preventing the spread of infectious diseases, saving lives, and ultimately promoting public health and well-being within society, has been well researched [2-7]. However, assessing unstructured user-generated content on social media can be time consuming [8], limiting the ability to harness the full potential of this approach to understand and improve public health. This has previously led researchers to use foundational methods, such as natural language processing (NLP), supervised machine learning, and other approaches to help interpret data [7,9,10]. For example, topic modeling or classification of posts can be used as an initial step before subsequent manual analyses, but even those methods can be inaccurate and time consuming, perhaps even more so for analysis of a larger corpus of text [8,11-16].

### Potential Public Health Role for Large Language Models

Recently, it has come to light that “few-shot” or “zero-shot” learners, such as generative large language models (LLMs), may have advantages for overcoming some of these limitations, including for extracting inference or reasoning from large corpora of text including health-related content, but with potential inherent bias and other concerns [17-22]. LLMs such as GPT4 (OpenAI Inc) that are based on a transformer architecture are neural networks trained on very large corpora of natural text [23,24].

### Traditional Social Media Analysis Challenges

Although manual inductive thematic analyses [9] and other similar manual approaches used in the literature are valuable for assessing unstructured and unlabeled social media content and depict themes of public interest, they demand an extensive burden of human time and effort for detailed content analysis by well-trained human subject matter experts. This makes it unfeasible to conduct large-scale, nearer real-time studies of social media listening to routinely inform public understanding and policy decisions, despite the time-sensitive nature and impact on public health of online discourses that constantly evolve during health emergencies [25,26]. Although it has been suggested LLMs have the potential for not only summarizing but also interpreting large amounts of text [18-20], it is not clear to what extent LLMs can analyze text to glean subtleties of health-related meaning and convey the resulting themes in a clear and detailed fashion. In the past, early LLMs had restricted context volume windows, making it difficult to conduct such analyses of large documents or corpora [27]. However, several newer LLMs have become available with an increased context window to allow analysis of larger documents and corpora, initially including GPT4 and Claude 2 (Anthropic PBC) [28,29].

The application of LLMs to public health social listening approaches may have the potential to help expedite the processes of social media thematic analyses and make it more efficient than tasking human subject matter experts [30-32]. However, different LLMs can exhibit different biases or capabilities [33-35], including hallucinations (false information resulting from the token-prediction algorithm) that have not been appropriately evaluated [36]. Specifically, there have not been abundant studies validating the use of LLMs for thematic analysis of large corpora of health-related social media content. It is foreseeable that public health and health care stakeholders are or will begin to more rapidly adopt LLMs to generate automated reports using large unstructured social media or similar health data sets [16,17,30]. Before assuming LLMs can achieve the equivalent of humans in the context of thematic identification or in-depth content coding, it is important to compare results from LLMs to those from human qualitative analysis on specific topics of public health importance, such as vaccine rhetoric [37,38]. The development of topic models is often misaligned with the needs of users who analyze social media data [39]. Evidence suggests that researchers frequently use topic models suboptimally because of a lack of adequate methodological support for building and interpreting topics [39]. This gap in support leaves researchers struggling to fully leverage topic models in their analyses [39].

### Study Purpose and Goals

In this comparative study, we evaluate the feasibility of using LLMs for topic model selection or inductive thematic analysis of health-related social media posts on vaccine rhetoric discourse [34,35]. We compare the output of 3 different LLMs to conduct the same analysis that members of our group had previously conducted in which they had used a combination of data mining, topic modeling, and manual content analysis in a prior published study examining vaccine rhetoric on Twitter. Here, we used the same corpus of social media content and guidelines for the LLM analysis as was conducted during human annotation in the prior study, and then we conducted a comparative analysis [9]. Using the results from that prior study as background, in this current LLM study, we asked the following question: Can LLMs conduct topic model selection and inductive analysis in a manner comparable to human performance, or at least reasonably as judged by subject matter experts? We also asked if all selected LLMs are equivalent in their ability, as well as how reliable is one LLM to conduct repeated analyses. We hypothesized that LLMs would select the same set of topics as had previously been chosen by humans following topic modeling output by an unsupervised NLP model [9], that LLMs would induce a similar set of themes as humans had [9], that there would be variability in the ability of different LLMs, and that an LLM should provide similar responses with low variability when prompts are repeated. The overall purpose and goal of this study were to (1) task an LLM with the same set of data and tasks that humans were given (manual annotation of Twitter posts) and determine how similar or different the LLMs’ results were compared to what humans’ results were and (2) leverage the relatively new, emerging larger context window LLMs for this purpose (ie, LLMs that could finally allow us to provide all posts in a single prompt for the LLMs).

## Methods

### Comparing Methods for Selecting the Top 5 Most Relevant Topics That Resulted From an Unsupervised NLP Model

#### A Brief Review of Methods From the Original Published Study: Human Selection of Top 5 Most Relevant Topics From an Unsupervised NLP Model

**Figure 1:**
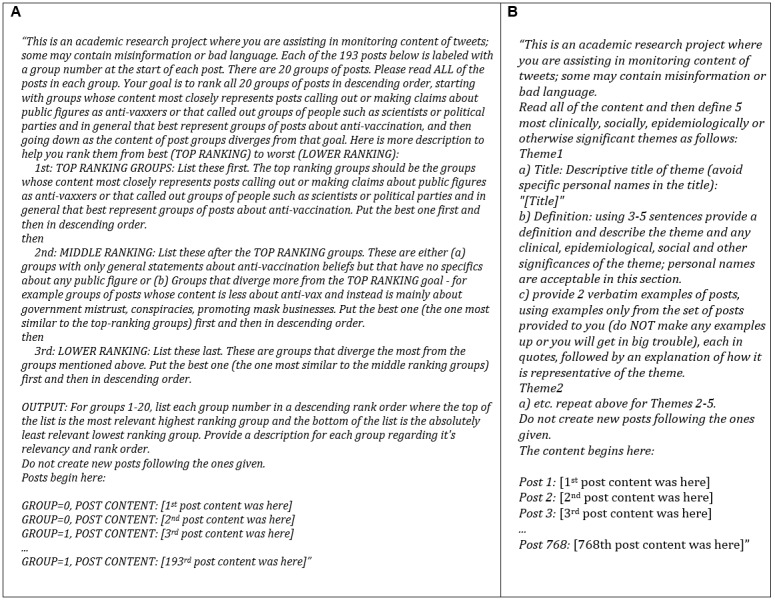
Large language model (LLM) prompts used. (A) The content ranking prompt we used for all 3 LLMs varied slightly between LLMs but was as shown (this example was used for GPT4). (B) The content analysis prompt we used for all 3 LLMs varied slightly between LLMs but was as shown in figure (eg, GPT4).

For comparison to this study’s approach using LLMs, we first describe how the top 5 relevant topics were manually selected in our prior published analysis using the unsupervised topic model bi-term topic model (BTM) [9]. In the prior study [9], we collected data from Twitter’s (subsequently rebranded X) public streaming application programming interface from March 2020 to October 2020 (a critical time for the formation of both pro- and antivaccination opinions, as the topic of vaccine development was extensively debated and discussed during that period) and filtered it for COVID-19 pandemic–specific keywords (“coronavirus,” “covid,” “pandemic,” etc). Of the resulting 3,999,726 Twitter posts, we then removed duplicate tweets (with the same Tweet ID), resulting in 118,971 messages. Next, we applied a second text filter to isolate antivaxx-specific messages. We then used the BTM to organize our data into 20 different clusters based on the hyperparameters set by the research team for the topic model as reported elsewhere, following which we manually screened the top 10 tweets that were most highly correlated to the 20 topic clusters [9]. Finally, using this set of top 10 tweets from 20 clusters, we identified the 5 BTM topics most relevant to our research question by manually identifying the 5 clusters that most closely included messages calling out or making claims about public figures that opposed vaccination or that called out groups of people, such as scientists or political parties. We chose to focus our analysis on public figures as they are highly influential in our society, especially on social media. We aimed to assess how their online presence and discourse affect public attitudes and sentiments toward health recommendations and policies. By focusing on public figures, we sought to understand the role they play in shaping public opinion and the potential impact of their statements on the dissemination of antivaccine messages (public figure names have been deidentified, and we have replaced them with generalized names in square brackets). The topics included [tennis pro]’s *antivaccination stance*; [public figure 1] *and* [philanthropist]’s *relation to antivaccination beliefs*; [politician 1]’s *potential antivaccination stance*; [politician 2] *and Amy Duncan (of note: Amy Duncan is a fictional character played by actress* [actress 1]); and *political party potential antivaccination views.* Although each topic comprised several tweets, our analysis focused solely on comparing the top 10 most relevant tweets from each cluster, enabling us to efficiently identify the 5 clusters and corresponding themes most pertinent to our research question [9]. Of note, the set of top 10 most correlated tweets from 20 clusters is the same set of posts that we then used in this study for LLM-based top 5 most relevant BTM topic selection, described in the following section.

#### LLM-Based Top 5 Most Relevant BTM Topic Selection

For this study, we sought to replicate the aforementioned manual BTM topic selection process of identifying the 5 clusters (that most closely included messages calling out or making claims about public figures as antivaxxers or that called out groups of people such as scientists or political parties). However, here, we used LLMs for this process in place of the previous, more manual approach. To do this, we first prepared the same set of posts (the original set of the top 10 tweets that were most highly correlated to the 20 topic clusters from the original manual study) for use with LLMs by labeling each post with an original BTM topic group ID of 1 to 20. This was to allow the LLM to know which BTM topic group each post was part of. We then asked the LLMs to rank the BTM groups from 1 to 20 in the order of relevance as related to the guidelines used by subject matter experts when they had manually selected the 5 most relevant topics in the prior publication. We then compared how well the top 5 (out of 20) topics ranked by LLMs compared with the 5 out of 20 topics previously chosen manually. Additional details were as follows: The LLMs and platforms we used were GPT4-32K, Claude-instant-100K, and Claude-2-100K, accessing them via the Poe [40] (Mountain View) platform. To use the Poe interface, we manually pasted in prompt texts and copied out the results; we refer to each of these events as “test runs” in the manuscript. For data, the original corpus of posts contained 193 posts, labeled with one of 20 original BTM topic numbers. This list of topic numbers and post content was included in the prompt shown in Figure 1A. The content ranking prompt we used for all 3 LLMs varied slightly between LLMs, but it was as shown in Figure 1A (this example was used for GPT4).

#### Statistical Assessment of the Top 5 Topics Ranked by LLMs

We tested the null hypothesis that the LLM’s top 5 BTM topic rankings out of 20 in this study would be independent of the top 5 BTM topics chosen in the previous study by the human raters. We modeled LLM choice under this null hypothesis as random sampling without replacement (ie, the number of topics chosen by the model that had been chosen by the human was assumed given by the hypergeometric distribution under the null hypothesis). We chose this approach because, under the assumption that the LLM picked choices randomly and independently of the human choices, the number of agreements with the human choices is given by the hypergeometric distribution. If the LLM agreed with the human more often than the hypergeometric would lead us to expect, we conclude the LLM is more likely to pick the human choices than chance alone would indicate. For each LLM, we first determined the number, N, of the human-chosen top 5 BTM topics that the LLM ranked as its top 5 topics (necessarily, N is in the range of 0-5). We then computed the probability using the hypergeometric distribution. Using the number of matches as a test statistic, the probability that the LLM would have picked as many or more of the human choices as we observed therefore provides a probability value as a way to assess that the LLM choices were unrelated to the human choices. In this way, the more of the original 5 BTM topics chosen by humans that were also ranked in the top 5 by LLMs, the lower the probability that the result was by chance alone. Therefore, a small probability value indicates that LLMs made topic choices similar to those of humans.

### Comparison of Inductive Thematic Analysis by Humans Versus by LLMs

#### Methods of Original Study, Human Inductive Thematic Analysis to Identify and Define 5 Themes

In comparison to this study’s approach using LLMs to automatically identify key themes based on the content of the posts, we first briefly describe how the top 5 themes were manually identified, selected, and defined in our initial published manual analysis [9]. In the previous paper, the human team used grounded theory, allowing for themes to emerge while coding rather than prespecifying the content of interest. After the first round of manual review, we inductively developed a codebook for the qualitative content analysis and categorization of Twitter posts. We then reapplied our codebook to the 768 Twitter messages in our sample while iteratively continuing to develop existing codes and definitions as well as new codes. Ultimately, from about 7 themes that we considered met our criteria (to identify the top clinical, social, epidemiological, or otherwise relevant themes), we selected 5 of them to narrow the focus and describe them in the manuscript:

- Theme A: Neutral—absence of expression of a clear judgment even if the message is related to the topic
- Theme B: Insults a person because they are an antivaxxer or says something derogatory to someone because they are or have been accused of being an antivaxxer
- Theme C: Negative public health impact—states or implies that antivaxxers and antivaccine behaviors have a negative impact on public health
- Theme D: Antivax accusation—accuses or asserts a specific person or groups of people are antivaxxers
- Theme E: Defending antivax stance—defends or upholds an antivax position.

These findings were important as they suggested a reciprocal influence between public health recommendations and attitudes toward public figures, challenging the previously described notion of a 1-way, outsized influence of celebrities on vaccination attitudes. This nuanced understanding of vaccine sentiment and its interplay with public figures challenged conventional narratives about the influence of celebrities on vaccination attitudes. It also highlighted the complex relationship between public health recommendations, societal perceptions of authority figures, and individual beliefs, underscoring the need for tailored interventions and messaging strategies. This social listening study provided insights into the dynamics of vaccine discourse on Twitter and informed on potential strategies for public health officials and policy makers to craft more effective communication strategies to promote vaccine acceptance and uptake. However, this manual human inductive thematic analysis process in our prior study took many days and hours of effort for all team members. Therefore, in this study, we sought to leverage LLMs to try to conduct the same analysis and assess the outcome (described in the subsequent section). Of note, the set of 768 Twitter messages in the published human assessment study is the same set of posts that we then used in this study for LLM inductive analysis to identify and define 5 themes with titles, definitions, and representative posts.

#### LLM Inductive Analysis to Identify and Define 5 Themes With Titles, Definitions, and Representative Posts

In this study, we have used the aforementioned original set of 768 Twitter messages from the published inductive thematic analysis manual study to include them in our LLM content analysis prompt shown below to prompt the LLM to deduce themes (a very similar prompt was used for all 3 LLMs). Using the prompt submission as shown in Figure 1B, results were obtained twice (ie, test runs 1 and 2) each for GPT4-32K, Claude-instant-100K, and Claude-2-100K. Each test run was independent of any other run. Completing these tasks took approximately 45 minutes of one researcher’s effort. The content analysis prompt we used for all 3 LLMs varied slightly between LLMs but was as shown in Figure 1B (this example was used for GPT4).

#### Assessing Hallucination (Generation of Phantom Posts) in Responses Given by LLMs

Before assessing the themes identified by the LLMs, we first reviewed the “example posts” provided in the LLM responses to assess how many of the social media post examples provided in the LLM responses were actually a part of the original 768 posts we had provided compared to how many post examples provided by the LLMs were “phantom posts” fabricated via hallucination by the LLMs and not in the original set of 768 posts we had provided the LLM. We assessed the accuracy of these example posts to determine if the LLMs generated phantom posts, ensuring that they identified themes accurately without altering the original post content. For each example post provided in each LLM response, we assessed its similarity to the original post from the prompt that had been presented to the LLM and classified it as an identical example post (a verbatim copy of a post from the original LLM prompt), near-identical example post (very similar to an original post in the LLM prompt but not completely identical such as a missing period or added number) or a phantom example post (the LLM provided us an example of an original post that was not obviously similar to any original post in the LLM prompt). We then summarized the results for each of these 3 categories overall and tabulated the totals by LLM platform and test run.

#### Assessing Themes in the Responses Given by LLMs

To assess the themes identified by the LLMs, 2 subject matter experts, who were the authors and manual annotators of the original manuscript, reviewed the themes identified by each LLM test-run output response. For each response, we identified (1) how many of the 5 themes provided matched the original manuscript themes, (2) which themes matched, and (3) how reasonable on a scale of 1to 3 was each derived theme, regardless of whether it matched the original manuscript theme. We evaluated the match between the original themes and the LLM-derived themes by assessing for relevance, accuracy, and fidelity to the original themes. We then reported the individual and total matches per LLM test run. For assessing reasonableness, each LLM-derived theme was scored by our subject matter expert team members from 1 to 3 where “1” meant not reasonable (the LLM theme matched poorly with little relevance to the original manuscript); “2” meant reasonable (the LLM theme matched moderately well, with some relevance to the original manuscript); and “3” meant very reasonable (the theme matched closely and accurately with high relevance to the original manuscript). We then averaged the scores of the 2 authors to assign a reasonableness score.

### Comparison of Inductive Thematic Analysis by Humans Versus Pseudoinductive Thematic Analysis Outputs From the Topic Models Latent Dirichlet Allocation and BERTopic

Finally, for comparison to the ability and utility of using LLMs for assessing themes, we also compared that to using 2 topic models to produce outputs from the 768 posts and assessing those outputs as we did for LLMs (did they match the original human themes and how reasonable are they), as well as an additional assessment to compare how clear the meaning of the outputs of the topic models was for humans compared to how clear in general the meaning of the outputs of the LLMs was for humans.

#### Topic Modeling and Producing Word Frequency Topic Grids for Human Assessment

Using the same set of original 768 posts we had provided, we used 2 topic modeling frameworks, latent Dirichlet allocation (LDA) and BERTopic, to develop topics that we then visualized for humans to assess.

LDA is a topic modeling technique to extract topics from a given set of texts; it converts the text into a bag of words and categorizes them into k different clusters based on their similarity. It then outputs the correlation score between each text and the cluster, with the correlation score between each word and cluster; the text or word that has a higher correlation score is more likely to contain topics related to the rest of the corpus. In our assessment, first, we extracted 5 topics from the data set, and for each, we output the top 20 keywords that have the highest correlation score to represent the topic for each cluster, and then output a list of text sorted based on the correlation score from each topic, for human assessment.

BERTopic is a topic modeling technique that leverages transformers and class-based term frequency–inverse document frequency to create dense clusters, allowing for easily interpretable topics while keeping important words in the topic descriptions [41]. Instead of converting each text into a bag of words, it uses a pretrained deep learning model and embeds each text into a text vector and then categorizes them using a clustering model. In our assessment, we used KMeans as our clustering model to avoid the output of outliers, set the minimum size of each cluster to 10, and then extracted 5 topics. For each topic, we then output the top 20 keywords with the highest correlation score from each cluster to represent the topic. However, because KMeans directly assigns each text to different topics, we were unable to obtain the text correlation score for BERTopic.

For visualization of the results from both LDA and BERTopic, we created topic grids. To create the topic grid for each topic, the top 20 keywords were included in cells within the grid. The background colors ranged from dark to light and font size ranged from largest to smallest, reflecting the correlation between each keyword and the topic, from the most to least relevant, respectively.

#### Developing and Assessing the Pseudothemes in the Outputs Provided by Topic Model Outputs

For LDA and BERTopic, a PDF file was produced with the topic grid and a list of word frequencies for each topic. These 10 topic model output pdf pages (1 per topic and 5 topics per topic model) were provided to one of the team members who had also assessed the output of the LLMs versus the original human themes. Unlike the LLM outputs, the topic models provide no theme title, no description of a theme, and no reasons for why any example posts support that theme, making it difficult to compare the topic model results to the themes of the original manuscript. Therefore, to assess the utility of using these topic model outputs for thematic induction, our team’s subject matter experts reviewed the 5 topic outputs from LDA and BERTopic and then manually developed a pseudotheme for each topic. For each, they reviewed the topic grid and list of word frequencies and then they manually developed a title for each topic, which we will refer to in this manuscript as the topic’s “pseudotheme.” Assessment of pseudotheme outputs was then conducted similar to the assessment of LLM outputs: for each topic model output (LDA and BERTopic), we identified (1) how many of the 5 topic pseudothemes matched the original manuscript themes; (2) which original manual theme they matched; and (3) how reasonable on a scale of 1 to 3 was each derived topic pseudotheme, despite whether it matched the original manuscript themes. We then reported the individual and total matches per topic model output.

As the topic model output grid was much less of a clear, comprehensive narrative than the output of the LLMs, for scoring matches to original themes, for assessing reasonableness, each topic pseudotheme was assigned a “reasonableness score” by our subject matter expert team members. Scores ranged from 1 to 3, where “1” meant not reasonable (the topic pseudotheme matched poorly with little relevance to the original manuscript); “2” meant reasonable (the topic pseudotheme matched moderately well, with some relevance to the original manuscript); and “3” meant very reasonable (the topic pseudotheme matched closely and accurately with high relevance to the theme of the original manuscript). This scoring process was repeated while masked to the first scoring round results. We then averaged the scores of the 2 rounds to assign a reasonableness score to each topic model’s pseudotheme.

Finally, as we noted potentially more difficulty for humans to interpret the topic model grid outputs compared to interpreting LLM outputs, we assigned a “clearness score” comparing our ability to understand the topic model output pseudotheme to how easily and quickly we confidently understand the meaning and theme of the typical LLMs theme outputs. We assigned a clearness score from 1 to 3 as “1” (this topic model result is much harder for me to easily, quickly, and confidently understand the meaning and theme of compared to the LLM outputs); “2” (this topic model result is about the same for me to easily, quickly, and confidently understand the meaning and theme of compared to the LLM outputs); and “3” (this topic model result is much easier for me to easily, quickly, and confidently understand the meaning and theme of compared to LLM outputs). This clearness scoring process was repeated while masked to the first scoring round results. We then averaged the scores of the 2 rounds to assign a clearness score for each topic model’s pseudotheme.

### Ethical Considerations

As this study used deidentified, publicly available social media data, the Institutional Review Board of University of California, San Francisco, classified our proposal as exempt from review (IRB 13-12815).

## Results

### Selecting the Top 5 Most Relevant Topics That Resulted From an Unsupervised NLP Model

#### Brief Review of Results From the Original Published Study: Human Selection of Top 5 Most Relevant Topics From an Unsupervised NLP Model

The 5 topics chosen by humans as the 5 most relevant topics in the published original manual analysis [9] are described in the methods and shown in Table 1 in the headers for columns 2 to 6.

**Table 1:** Relevance ranking (out of 20 BTM^a^ topic groups) by LLMs^b^.

LLMs^c^			BTM topic											Hypergeometric probability^d^
			[Tennis pro] antivaxxer stance^e^		[Public figure 1] or [philanthropist] relation to antivax beliefs^e^		[Politician 1] potential antivaxxer stance^e^		Political party potential antivax views^e^		Amy Duncan (actress: [actress 1]), [politician 2]^e^			
**GPT4**														
	First test run^f^	1		2		3		6		19		.07		
	Second test run^f^	1		2		3		10		16		.07		
	Third test run^f^	1		2		3		5		19		.05		
**Claude 1**														
	First test run	1		6		5		4		12		.07		
	Second test run^f^	1		5		4		11		18		.07		
	Third test run^f^	1		19		2		10		16		.37		
**Claude 2**														
	First test run	1		8		12		4		17		.37		
	Second test run^f^	1		5		4		11		18		.07		
	Third test run^f^	1		19		2		10		16		.37		

^a^BTM: bi-term topic model.

^b^LLM: large language model.

^c^LLM platform and test run number.

^d^Looking at the top 5 topics selected by the LLM test run in that row, this column shows the probability that by chance alone, we would have seen as many or more matches of the LLM-chosen top 5 with the 5 chosen by humans, compared with what we actually observed. The hypergeometric probability shows the probability that the LLM would agree with as many or more of the human choices by chance alone.

^e^Top 5 most relevant topics (out of 20 BTM topic groups) assigned by human raters in the original manuscript.

^f^Rows: Each row contains the results of an LLM test run to assess the corpus of 193 posts, each of which was labeled in the original manuscript BTM methods as being from one of the 20 BTM topics. For each row: each cell shows the ranking (1 most relevant; 20 least relevant) assigned by the LLM for the original topic in the header of that cell’s column (ie, for the original manuscript’s topic shown in the header of that cell’s column).

#### LLM-Based Top 5 Most Relevant BTM Topic Selection

We obtained results from each LLM using 3 test runs per LLM. Completing these tasks took approximately 1 hour of researcher effort. Each LLM was able to assign rank orders to the 20 topics. In Table 1 for each LLM analysis (each LLM row), columns 2 to 6 show the relevance ranking (1 being most relevant and 20 being least relevant) assigned by the LLM for each of the 5 original topics chosen by humans in the original manuscript. Overall, the results suggest LLMs make many of the same 5 topic choices as humans did; GPT was the most successful, followed by Claude 1.

#### Statistical Assessment of the Top 5 Topics Ranked by LLMs

Table 1 shows the names of the top 5 most relevant topics that were chosen manually in the original manuscript [9] and the rankings (out of 20) assigned by the LLM for each of those 5 original topics. When comparing how many of the LLM’s top 5 ranked topics were the same 5 topics from the manuscript, different LLMs yielded different results. GPT-4’s top 5 ranked topics in test runs included 3 or 4 of the 5 topics from the original manuscript, with a mean of 3.3 (SD 0.58) over 3 runs. Claude 1’s top 5 ranked topics in test runs included 2 or 3 of the 5 from the manuscript with a mean of 2.7 (SD 0.58), whereas Claude 2’s top 5 ranked topics in test runs included between 2 and 3 of the 5 from the manuscript with a mean of 2.3 (SD 0.58). When broadening to include the LLM’s top 10 ranked (rather than just the top 5 ranked) topics for comparison to the 5 topics from the manuscript, the 3 GPT’s top 10 ranked topics in test runs included 4 of the 5 topics from the manuscript with a mean of 4.0 (SD 0.0), the Claude 1’s top 10 ranked topics in test runs included 3 or 4 of the 5 from the manuscript with a mean of 3.67 (SD 0.58), and finally the Claude 2’s top 10 ranked topics in test runs included 2 or 3 of the 5 from the manuscript with a mean of 3.0 (SD 0.0).

The overall result for each of the 9 LLM test runs conducted (each row) is in Table 1, hypergeometric probability column. Values shown represent the probability that by chance alone, we would have seen as many or more matches of the LLM-chosen top 5 with the 5 chosen by humans, compared to what we actually observed in that test run. A low probability is evidence that the LLM’s choices agree with those chosen by humans more than expected by chance alone. Combining all 9 test run results, testing the hypothesis that all LLMs were independent of each other and the humans, we rejected this null hypothesis (*P*<.001, overall comparison; Monte Carlo: N=16,384), and conclude that the LLMs are more likely to include the top human 5 groups in their top rankings than would occur by chance.

### Comparison of Inductive Thematic Analysis by Humans Versus LLMs

#### Brief Results of Original Published Study: Human Inductive Thematic Analysis to Identify and Define 5 Themes

The 5 themes identified and defined by humans in the published original manual analysis [9] are described in the *Methods* section.

#### LLM Inductive Analysis to Identify and Define 5 Themes With Titles, Definitions, and Representative Posts

In general, all LLMs were provided responses that were outputted in the requested format. An example of output for GPT-4 32k is shown below in Textbox 1, and additional examples for Claude are shown in Textboxes S1 and S2 in Multimedia Appendix 1.

**Example of GPT-4 32 K inductive thematic analysis. The prompt asked the large language models (LLM) to read 768 posts and define themes, including a title, description, and post examples. GPT4 test run 1 output is shown.**

**Theme 1**

1. Title: Public Perception and Discussion of Celebrities as Anti-Vaxxers
2. Definition: this theme captures discussions around public figures, notably celebrities, such as athletes and actors, being perceived as antivaxxers. The public’s reaction to these figures espousing antivaccination views can influence societal attitudes toward vaccination, potentially impacting public health initiatives and vaccination rates. In addition, this theme showcases the role of social media in rapidly disseminating and amplifying these views.
3. Examples: “Post 6: [tennis pro] is an anti-vaxxer cool cool cool.” This post highlights a casual reaction to the news about a celebrity being an antivaxxer, indicating how such information can be normalized within social networks. “Post 80: Wow. I had no idea that [tennis pro] is an anti-vaxxer.” This post exemplifies the surprise or shock that can occur upon learning a well-known figure holds antivaccination beliefs. This can prompt further discussion and sharing of the information.

**Theme 2**

1. Title: Expressions of Disappointment and Betrayal Toward Anti-Vaxxer Celebrities
2. Definition: this theme involves posts expressing disappointment, surprise, or a sense of betrayal upon learning that admired public figures are antivaxxers. This reaction can reflect the personal connection fans feel to celebrities and the influence those public figures can have on their fans’ beliefs and behaviors.
3. Examples: “Post 77: Oh [tennis pro]! I was such a fan 
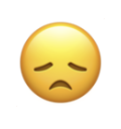
 #antiantivacc Coronavirus: [tennis pro] reveals he’s an anti-vaxxer and it may stop his return to tennis | World News | Sky News.” This post exemplifies the disappointment fans can feel when they learn a celebrity they admire holds views they disagree with. Post 97: [tennis pro] being an anti-vaxxer... i guess i have to stan [tennis pro 2] now.” This post shows the potential for such revelations to shift fan allegiances and influence public opinion about celebrities.

**Theme 3**

1. Title: Concerns About the Impact of Celebrity Anti-Vaxxers on Public Health
2. Definition: this theme involves posts expressing concern about the potential public health impacts of celebrities espousing antivaccination views. These posts reflect an understanding of the influence celebrities can have on public opinion and behavior and the potential consequences for vaccination rates and public health.
3. Examples: “Post (18) In other news [tennis pro] is an anti-vaxxer on top of being an opponent of equal prize money for women tennis players. Puts me more firmly in [tennis pro 2] corner.” This post connects the antivaccination stance with other controversial views, suggesting a broader concern about the influence of such public figures. “Post (97) [tennis pro] being an anti-vaxxer... i guess i have to stan [tennis pro 2] now.” This post highlights the potential for celebrity antivaxxer views to influence public opinion and behavior, including decisions about vaccination.

**Theme 4**

1. Title: Expression of Negative Sentiments Toward Anti-Vaxxer Celebrities
2. Definition: this theme involves posts expressing strong negative sentiments toward celebrities who are antivaxxers. These posts often include harsh language and criticism, reflecting strong feelings of opposition toward the antivaccination stance.
3. Examples: “Post 17: He’s an anti-vaxxer? Fuck [tennis pro].” This post reflects strong negative sentiments toward the celebrity, highlighting the emotional intensity that discussions around vaccination can provoke. “Post 101: idiot. [tennis pro] reveals he’s an anti-vaxxer and it may stop his return to tennis.” This post uses derogatory language to express disapproval of the celebrity’s antivaccination stance, reflecting strong opposition to such views.

**Theme 5**

1. Title: Potential Consequences of Anti-Vaxxer Beliefs for Celebrities’ Careers
2. Definition: this theme involves posts discussing the potential impact of antivaccination beliefs on celebrities’ professional careers. This includes speculation about the potential loss of opportunities or negative impacts on their public image.
3. Examples: “Post (19) #[tennis pro] is #antivax [tennis pro]’s opposition to vaccination may stop his return to tennis.” This post reflects concerns that antivaxxer beliefs could have practical consequences for the celebrity’s career, in this case, potentially preventing a return to professional tennis. “Post (66) Coronavirus: [tennis pro] reveals he’s an anti-vaxxer and it may stop his return to tennis. Twat.” This post combines negative sentiment toward the celebrity with speculation about potential career impacts, highlighting the perceived seriousness of their antivaccination stance.

#### Assessing Hallucination (Generation of Phantom Posts) in Responses Given by LLMs

Overall, the LLMs rarely produced phantom examples of original posts when providing post examples in their responses (2/60) and only by Claude 1. All other example posts provided by LLMs were identical (47/60) or near identical (11/60) to the original posts provided in the prompt. In general, GPT-4 performed the best (19/20 identical; 1/20 near identical) compared with Claude 1 and Claude 2. Broken down by LLM platform and test run, the results are shown in Table 2.

**Table 2:** Assessment of hallucination (generation of phantom posts) in responses given by LLMs^a^ relevance ranking (out of 20 BTM^b^ topic groups) by LLMs.

LLMs		Identical example post	Near-identical example post	Phantom example post
**GPT4**				
	First test run^c^	10/10	0/10	0/10
	Second test run^c^	9/10	1/10	0/10
**Claude 1**				
	First test run^c^	6/10	3/10	1/10
	Second test run^c^	7/10	2/10	1/10
**Claude 2**				
	First test run^c^	8/10	2/10	0/10
	Second test run^c^	7/10	3/10	0/10

^a^LLM: large language model.

^b^BTM: bi-term topic model.

^c^Each row shows the results of an LLM test run for which each of the 10 example posts provided by a given LLM response was compared with the original pool of posts presented to the LLM in the prompt and then classified as an: identical example post (a verbatim copy of a post from the original LLM prompt), near-identical example post (very similar to an original post in the LLM prompt, but not completely identical, such as a missing period or added number) or a phantom example post (the LLM provided us an example of an original post that was not obviously similar to any original post in the LLM prompt). The results for each of these 3 categories are tabulated by LLM platform and test run.

#### Assessing Themes in the Responses Given by LLMs

We first assessed how many of the themes identified by LLMs were equivalent to the themes from the original manuscript [9]. Overall our team’s 2 subject matter experts found that the inducted themes output by LLMs partially matched the 5 themes described in the manuscript [9]. Each human inductive analysis derived theme in the original manuscript was matched at least once successfully by an LLM test run, with the exception of the neutral category, which did not yield any corresponding matches. Table 3 shows the results for each LLM test run as compared to the original themes from the human thematic induction paper. In each cell the theme title provided by the LLM output is in quotes, and in each cell above the LLM theme title, we have indicated if the LLM theme matched one of the original paper’s themes A-E [9] or if there was no match with any of the original paper’s themes. Claude 1 most closely matched the themes from the original human inductive thematic analysis but did not identify every theme and was closely followed by outputs of both Claude 2 test run 1 and GPT test run 1. The GPT output from test run 2 only identified one theme, and both Claude 1 test run 1 and Claude 2 test run 2 outputs did not successfully identify any of the original themes.

Next, we assessed how reasonable each theme derived from each LLM test run was (independently of whether it matched a theme from the original human study). Our team’s 2 human subject experts determined that most of the LLMs themes were reasonable but varied by LLM. In Table 4, each cell includes the reasonableness score of an LLM’s theme, as an average of the 2 scores assigned by the 2 human assessors. As described in detail in the methods, scores ranging from 1 to 3 (1=not reasonable, 2=reasonable, and 3=very reasonable) were assigned to each theme in each test run. The average of all the scores assigned for a given test run are in the final column, and these ranged from 1.8 to 2.8. Reasonableness for each of the themes included in GPT test run 1 ranged from 2.5 to 3.0, whereas GPT test run 2 performed slightly worse. Themes included in Claude 1 test run 1 ranged from scores of 2-3, whereas the Claude 2 test run 1 performed relatively well, with the exception of a single theme that was determined to be a poor match. Both Claude 1 test run 2 and Claude 2 test run 2 performed relatively poorly, similar to how they had underperformed producing themes that matched the originals. Notably, both the matched themes and reasonableness were inconsistent between the 2 test runs for each given LLM.

**Table 3:** Original human-inducted themes and matches with LLM^a^-inducted themes or topic model pseudothemes^b^.

Source of the themes^c,d^	Inducted themes from original manuscript, from LLM test runs, or topic models. For the LLM rows: *matches to original themes (A-E*) “LLM theme title”						Number match to human’s themes
	Theme A: neutral—*absence of expression of a clear judgment even if the message is related to the topic*^e^	Theme B: insults a person because they are an antivaxxer—*says something derogatory to someone because they are or have been accused of being an antivaxxer*^e^	Theme C: negative public health impact*—states or implies that antivaxxers and antivaccine behaviors have a negative impact on public health*^e^	Theme D: antivax accusation—*accuses or asserts a specific person or groups of people are antivaxxers*^e^	Theme E: defending antivax stance— *defends or upholds an antivax position*^e^		
GPT4, first test run^f^	(No match) “Public Perception and Discussion of Celebrities as Anti- Vaxxers”	Match: Theme B “Expressions of Disappointment and Betrayal Toward Anti-Vaxxer Celebrities”	Match: Theme C “Concerns About the Impact of Celebrity Anti-Vaxxers on Public Health”	Match: Theme B “Expression of Negative Sentiments Toward Anti-Vaxxer Celebrities”	(No match) “Potential Consequences of Anti-Vaxxer Beliefs for Celebrities’ Careers”	3/5	
GPT4, second test run^f^	(No match) “Public Perception of Celebrities and Vaccination Stances”	(No match) “Emotional Responses to Anti-vaccination Views”	(No match) “Public Criticism and Condemnation of Anti-Vaccination Views”	(No match) “Potential Consequences of Anti-Vaccination Views”	Match: Theme B “Public Shaming and Ridicule of Anti-Vaccination Views”	1/5	
Claude 1, first test run^f^	Match: Theme E “Vaccine skepticism during the COVID-19 pandemic”	Match: Theme D “Accusations of being ‘anti-vaxxers’ in the political discourse”	Match: Theme B “Negative reactions to celebrity anti-vaccine stances”	(No match) “Comparisons between anti-lockdown and anti-vaccine movements”	Match: Theme C “Spread of anti-vaccine messaging during the pandemic”	4/5	
Claude 1, second test run^f^	(No match) “Anti-vaxxer sentiment”	(No match) “Debate over COVID-19 vaccines”	(No match) “COVID-19 vaccine promotion and misinformation”	(No match) “Lockdown and public health protest activity”	(No match) “Popular culture, celebrities, and public discussions”	0/5	
Claude 2, first test run^f^	Match: Theme E “Skepticism toward COVID-19 vaccines”	Match: Theme C “Blaming deaths and outbreaks on anti-vaccine views”	(No match) “[Tennis Pro]’s COVID-19 diagnosis”	Match: Theme B “Insults and criticisms of anti-vaccine people”	(No match) “Anti-vaccine views linked to other conspiracies”	3/5	
Claude 2, second test run^f^	(No match) “Anti-vaxxer sentiment”	(No match) “Politicization of vaccines”	(No match) “Vaccine misinformation”	(No match) “Vaccine hesitancy”	(No match) “Calls for vaccination”	0/5	
LDA^f,g^	(No match) “Protesting lockdowns”	(No match) “[Tennis Pro] with COVID-19”	(No match) “Distrusting politicians and COVID vaccine”	(No match) “Numerous themes”	(No match) “Anti-vaccination COVID conspiracies”	0/5	
BERTopic^f,h^	(No match) “Anti-vaccination and anti-lockdown”	(No match) “Distrust in the COVID-19 vaccine, and politicians”	(No match) “[Tennis Pro] anti-vaxxer stance”	(No match) “Anti-vaccination conspiracies”	(No match) “Anti-lockdown opinions and protests”	0/5	

^a^LLM: large language model.

^b^Shows the results for each LLM test run (or topic model output) compared with the original themes from the human thematic induction paper.

^c^Column 1 indicates the source, for each row, that leads to the themes provided in that row.

^d^Humans (themes A-E are from inductive analysis–derived themes of the original manuscript).

^e^Five LLM-inducted theme titles from the LLM’s output for that test run are shown in columns 2-6, in quotes. In each cell above the LLM theme title, we have indicated if the LLM theme (or topic model pseudotheme) matched to 1 of the original paper’s themes (A-E) and if so, we indicated the theme it matched. For LLMs or topic models in which there was no match with any of the original paper’s themes this is indicated with “(no match).”

^f^Subsequent rows indicate the LLM test run (or topic model output) in this study used to derive themes from those same 768 posts.

^g^LDA: latent Dirichlet allocation.

^h^BERTopic: Bidirectional Encoder Representations from Transformers with class-based term frequency–inverse document frequency.

**Table 4:** Reasonableness of LLM^a^-inducted themes or topic model pseudothemes^b^.

Source	LLM-inducted theme titles and subject matter expert assigned reasonableness scores (and clearness scores for LDA^c^ and BERTopic^d^)						Scores^e^, mean (SD)
	Title 1	Title 2	Title 3	Title 4	Title 5		
GPT4, first test run^b,f^	“Public Perception and Discussion of Celebrities as Anti- Vaxxers” (reasonable, 2.5)^e,g^	“Expressions of Disappointment and Betrayal Toward Anti-Vaxxer Celebrities” (reasonable, 3) ^e,g^	“Concerns About the Impact of Celebrity Anti-Vaxxers on Public Health” (reasonable, 3) ^e,g^	“Expression of Negative Sentiments Toward Anti-Vaxxer Celebrities” (reasonable, 3) ^e,g^	“Potential Consequences of Anti-Vaxxer Beliefs for Celebrities’ Careers” (reasonable, 2.5) ^e,g^	R: 2.8 (0.27) ^i^	
GPT4, second test run^b,f^	“Public Perception of Celebrities and Vaccination Stances” (reasonable, 2.5) ^e,g^	“Emotional Responses to Anti-vaccination Views” (reasonable, 2.5) ^e,g^	“Public Criticism and Condemnation of Anti-Vaccination Views” (reasonable, 3) ^e,g^	“Potential Consequences of Anti-Vaccination Views” (reasonable, 2) ^e,g^	“Public Shaming and Ridicule of Anti-Vaccination Views” (reasonable, 2.5) ^e,g^	R: 2.5 (0.35) ^i^	
Claude 1, first test run^b,f^	“Vaccine skepticism during the COVID-19 pandemic” (reasonable, 2.5) ^e,g^	“Accusations of being ‘anti-vaxxers’ in the political discourse” (reasonable, 3) ^e,g^	“Negative reactions to celebrity anti-vaccine stances” (reasonable, 3) ^e,g^	“Comparisons between anti-lockdown and anti-vaccine movements” (reasonable, 2) ^e,g^	“Spread of anti-vaccine messaging during the pandemic” (reasonable, 2.5) ^e,g^	R: 2.6 (0.42) ^i^	
Claude 1, second test run^b,f^	“Anti-vaxxer sentiment” (reasonable, 2) ^e,g^	“Debate over COVID-19 vaccines” (reasonable, 2.5) ^e,g^	“COVID-19 vaccine promotion and misinformation” (reasonable, 1.5) ^e,g^	“Lockdown and public health protest activity” (reasonable, 1.5) ^e,g^	“Popular culture, celebrities, and public discussions” (reasonable, 2) ^e,g^	R: 1.9 (0.42) ^i^	
Claude 2, first test run^b,f^	“Skepticism toward COVID-19 vaccines” (reasonable, 3) ^e,g^	“Blaming deaths and outbreaks on anti-vaccine views” (reasonable, 3) ^e,g^	“[Tennis Pro]’s COVID-19 diagnosis” (reasonable, 2.5) ^e,g^	“Insults and criticisms of anti-vaccine people” (reasonable, 3) ^e,g^	“Anti-vaccine views linked to other conspiracies” (reasonable, 1.5) ^e,g^	R: 2.6 (0.65) ^i^	
Claude 2, second test run^b,f^	“Anti-vaxxer sentiment” (reasonable, 2) ^e,g^	“Politicization of vaccines” (reasonable, 1.5) ^e,g^	“Vaccine misinformation” (reasonable, 1.5) ^e,g^	“Vaccine hesitancy” (reasonable, 2) ^e,g^	“Calls for vaccination” (reasonable, 2) ^e,g^	R: 1.8 (0.27) ^i^	
LDA^b,f^	“Protesting lockdowns” (reasonable, 2.5; clearness, 1.5) ^e,g,h^	“[Tennis Pro] with COVID-19” (reasonable, 2.5; clearness, 1.5) ^e,g,h^	“Distrusting politicians and COVID vaccine” (reasonable, 2; clearness, 1.5) ^e,g,h^	“Numerous themes” (reasonable, 2; clearness, 2) ^e,g,h^	“Anti-vaccination COVID conspiracies” (reasonable, 2.5; clearness, 1.5) ^e,g,h^	R: 2.3 (0.27); C: 1.6 (0.22) ^i^	
BERtopic^b,f^	“Anti-vaccination and anti-lockdown” (reasonable, 1.5; clearness, 1.5) ^e,g,h^	“Distrust in the COVID-19 vaccine, and politicians” (reasonable, 2; clearness, 2) ^e,g,h^	“[Tennis Pro] anti-vaxxer stance” (reasonable, 2.5; clearness, 3) ^e,g,h^	“Anti-vaccination conspiracies” (reasonable, 2; clearness, 1) ^e,g,h^	“Anti-lockdown opinions and protests” (reasonable, 1.5; clearness, 1) ^e,g,h^	R: 1.9 (0.42); C: 1.7 (0.84) ^i^	

^a^LLM: large language model.

^b^Test runs and LLM-inducted themes (or topic model pseudothemes) are the same as that shown in Table 3.

^c^LDA: latent Dirichlet allocation.

^d^BERTopic: Bidirectional Encoder Representations from Transformers with class-based term frequency–inverse document frequency.

^e^A reasonableness score, shown in parentheses in columns 2-6, is the average of scores assigned by the 2 human assessors of the LLM theme (or of the 2 pseudotheme assessments of the topic model outputs) on the basis of a scale of 0-3 (0=not understandable, 1=not reasonable, 2=reasonable, and 3=very reasonable).

^f^Each row represents a test run with column 1 indicating the LLM or topic model source.

^g^Columns 2-6 display the LLM-inducted theme titles (in quotes).

^h^For topic models rows, a clearness score is also provided (scored as, when compared with the LLM outputs, the ability to easily and quickly confidently understand the meaning and theme of the topic model output was as follows: 1, much harder than LLMs; 2, about the same as LLMs; 3, much easier than LLMs).

^i^Column 7 shows the average and SD of the column 2-6 scores for that LLM (or topic model) row, where “R” (all rows) is the mean reasonableness score for that row, and “C” (topic model rows) is the mean clearness score for the topic model outputs compared with that of LLM outputs.

#### Assessing Pseudothemes in the Outputs Provided by Topic Models

Regarding matches to the 5 human induced themes described in the original manuscript, overall our team’s 2 subject matter experts found that the inducted themes output by topic models never matched any of the original 5 themes (0/10, see LDA and BERTopic rows in Table 3). Regarding reasonableness, the topic model pseudothemes had scores comparable to the lower-performing LLMs (see LDA and BERTopic rows in Table 4). Regarding being understandable, the mean clearness score (see LDA and BERTopic rows in Table 4) reflected that it was more difficult to easily and quickly confidently understand the meaning and theme of each topic model output compared to the output of LLMs (1.6 for LDA and 1.7 for BERTopic, where 1=output is much harder than LLMs, 2=about the same as LLMs, or 3=much easier than LLMs).

## Discussion

### Principal Findings

Our principal findings compared to our original research questions and hypotheses are described here overall and then in further detail in subsequent sections. In this study, we asked if LLMs can conduct topic model selection from an analysis of a large corpus of health-related social media posts, equivalent to how humans did. We hypothesized that LLMs would select the same set of 5 most relevant BTM topics (out of 20) as had previously been chosen by humans. Overall, we have found that all LLMs studied could assess the large corpus of social media posts, provide outputs, and that some of these outputs identified the top 5 most relevant topic models compared to humans quite well. For example, the relevancy of BTM topic “[tennis pro] antivaxxer stance” was ranked number 1 by all LLM test runs and the relevancy of BTM topic “[politician 1] potential anti-vaxxer stance” was ranked in the top 5 by 8 of 9 LLM test runs. One particular original top 5 theme, BTM topic “Amy Duncan (actress: [actress 1]), [politician 2],” was consistently deemed not relevant by LLMs (most likely because of being about a fictional character, discussed in the Limitations section).

We also asked if LLMs can conduct inductive thematic analysis of a large corpus of health-related social media posts, equivalent to how humans did using the same corpus of 768 posts. We hypothesized that LLMs would induce a similar set of themes as humans had. Overall, we found that LLMs in our study identified several of the original themes identified by humans, with generally very low hallucination rates (almost no phantom posts were created in LLM responses). For example, Claude 2 identified a theme titled “Insults and criticisms of anti-vaccine people” with 0/10 phantom examples of original posts when providing post examples in its response and we determined this LLM theme was a match for the original human-inducted Theme B: “Insults a person because they are an anti-vaxxer; says something derogatory to someone because they are or have been accused of being an anti-vaxxer*.*” Our findings add to a growing body of literature in which LLMs are observed to provide similar (or at least reasonable) results to those provided by human assessors of a corpus of social media texts. For example, a recent study of topic model detection from news stories by humans compared to topic model detection by LLM found only minor variations in their respective topic evaluation scores and found GPT-4 outperformed other LLMs in their study [38], similar to its performance in our analysis. However, we did observe however that human coding appeared to have uncovered more depth and nuance, including that many posts were not amenable to a clear pro- or anti-vax stance, for example, a post such as, “Maybe I’m an anti-vaxxer because no no no no no waaaaaaaayyyyyy.” Future studies might investigate a hybrid approach as suggested by Haupt et al [16], in which a small subset of messages is coded by humans to potentially assist the LLM in detecting prominent themes and narratives within large corpora with improved depth and nuance.

In addition, we had hypothesized that even if not identical to the themes determined by humans, the LLMs’ assessment of the original 768 posts would at least produce reasonable themes, as judged by subject matter experts. Overall, we found that despite not consistently matching the original themes, many of the unmatched themes generated by the LLMs were still quite reasonable and relevant. For example, GPT 4 test run 2 only resulted in 1 theme that matched the original 5 human-determined themes; however, all the themes it provided were rated by our subject matter experts with reasonableness scores ranging from 2 (reasonable) to 3 (very reasonable), with an overall average score of 2.5.

We also asked if all LLMs are equivalent in their ability and had hypothesized that there would be variation in the ability of different LLMs. Our results demonstrated some variation between LLMs in ranking of the 20 BTM topics and in the themes generated by different LLMs consistent with our hypothesis and with the well-known observation that different LLMs can yield substantially different performance even with the same size-class [28,29,42], with some LLMs identifying more of the original themes than others.

Finally, we had hypothesized that any given LLM would provide similar responses with low variability when test run prompts are repeated. However, when using the same prompt with the same LLM, we found significant variation between test runs.

Overall, all of our results suggest that the utility of using the LLMs in our study for thematic analyses may be an efficient starting point, but do not currently match the ranking and especially the themes produced by a group of human subject matter experts that undertake in-depth qualitative content coding.

In our use of topic models as a comparator to using LLMs, we found that use of topic models (rather than LLMs) to attempt to extract a pseudotheme resulted in less effective matching of the original human themes (0/10). The topic models output’s pseudothemes had scores comparable to the lower-performing LLMs regarding being reasonable based on the content of the corpus of posts. In addition, the topic model pseudothemes were more unclear than the outputs from LLMs, and contained much less detail (no theme title, no description of the theme, no description of why examples of posts represented the theme), requiring additional subject matter expertise to interpret theme titles and pseudothemes from the topic model outputs, compared to LLM outputs.

### Relationship to Other Work

This study serves as a direct follow-up to our initial unsupervised topic modeling and manual content annotation social listening study of Twitter data, aiming to explore the potential expansion and optimization of this field through the use of LLMs. Previous research [43] has examined the role of social media in medicine and health care. This study contributes evidence of the utility of LLMs in conducing such research, and adds to the literature seeking to validate the use of LLMs, which is an evolving field [17,30,38].

### Limitations and Discussion of Less Successful Results

Despite fairly reasonable LLM results compared with humans in this study, some results were not consistent with the human-derived topics or themes, particularly for outputs generated by LDA and BERTopic. Exploring those differences can help us to improve performance in future studies or understand the limitations of our approach. For example, although many of the LLM rankings of the 5 most relevant BTM topics compared well to humans, the topic of “Amy Duncan (actress: [actress 1])” was consistently ranked in the bottom quartile by LLMs (see column 6 in Table 1). To understand the cause of this, we noted that in the prior study, to ensure relevance to public discourse, we had manually selected clusters containing both verified and unverified Twitter accounts of public figures and groups, and therefore this particular BTM topic that we had manually selected had content regarding fictional characters. It is likely the LLMs recognized the content of this BTM topic as being about a fictional character (ie, Amy Duncan), and, therefore, it may have ranked this topic low, as it is not about an actual public figure. This example demonstrates how humans may approach a task assessing a large corpus of social media posts differently than LLMs without very specific guidance to LLMs, and is an example of how iterative validations could help to improve the precision of an LLM prompt.

We also found that, despite decent performance by some of the LLMs, none of the LLMs actually generated themes that completely matched all 5 of the themes from the original analysis (Table 3) despite the use of very specific prompts that attempted to replicate the methods used for manual annotation in the original paper by Honcharov et al [9]. We note that for a task, such as thematic induction, there is always some level of subjectivity, even in our prior manual study. When we went back and reviewed the original study, we noted that the initial manual analysis of the data set had unveiled several supplementary key themes that were not incorporated into the report because of less overall agreement or difference in specific focus of the themes. Hence, future studies should investigate whether the LLMs would have identified similar supplementary themes if directed to do so and how this may differ based on the different specificity of prompts for topic modeling-related tasks requested of the LLMs. Therefore, these results might not be entirely surprising as they suggest that just as with humans, LLMs can exhibit subjectivity in interpreting a large corpus of content, resulting in variation in results. This concept remains open for exploration in future analyses.

We observed significant variation between repeat runs of an identical prompt with the same content and same LLM, as expected over a web-based interface in which it is not possible to set the temperature. In principle, the choice of temperature 0 should make the inference largely (though not perfectly) repeatable [44], but such a setting was not possible using a web-based chat interface. Further work is needed to determine whether multiple runs at a larger temperature setting achieves greater flexibility than a single run at 0 temperature. This variation also suggests the need for additional validation approaches that should be assessed through human supervision. Perhaps, LLMs could initially assess the outputs from multiple repeated test runs to assign a score of consistency between outputs, indicating areas with significant unexpected test-retest variation for follow-up human supervision.

Other limitations to our study include the fact that we only used X (Twitter) content, we focused just on vaccine-related content, and we did not use all available LLMs. All of these limitations can be addressed in future comparative analysis studies to help draw more broad conclusions about the acceptable use of our approach for other content sources, health topics, and different LLM platforms. In addition, we note that our analyses could not be fully masked, as original authors from the prior study conducted the assessment of LLM themes in this study.

### Future Studies and Potential Future Significance

Future human-LLM comparative studies on larger data sets and diverse social media corpora are needed to support our current findings before concluding that LLMs are a valid social listening tool to distill useful, relevant, unbiased and unhallucinated themes. Researchers in other health science domains should further examine LLMs to assess large corpora of their social media posts and different prompts with varying specificity to topic modeling tasks to accurately choose relevant topics and to describe main themes for other health topics of interest as these LLMs may be more fine-tuned for vaccine or misinformation-related thematic detection. This can be done using results from additional prior manual inductive thematic analysis studies and comparing the original manually derived results to that of LLMs as conducted in this study. Ideally, such studies would be conducted for any particular health science field before assuming results from one field are sufficient for another.

Future studies also may help to further assess the utility of variability even between results of repeat test runs for a given LLM (see the Limitations section). Although variability can be mitigated in LLMs by setting the LLM temperature parameter to 0, the variability may prove useful in deriving an ensemble thematic analysis, for potential increased performance (as is well-known for ensemble models in other fields) [44-46]. Although the human effort and time needed to complete the tasks in our study was a fraction of the human hours of time that the original manual study took (several hours in this study instead of ≥40 hours in the original study), future studies would be needed specifically to measure, compare, and substantiate claims of time savings, efficiency, and costs savings of using LLMs for health-related social listening.

Once validated, LLMs could find numerous social listening applications, including for disease forecasting (prediction) and nowcasting (providing data for situational awareness on what the public does, knows, or feels about health issues), data classification of established online health discourse topics and possible detection of new themes or trends, and efficiently grouping intersecting online health behavior queues and information seeking behavior for different health topics [47]. These applications could inform public health understanding of public interests and concerns, and to learn the public’s ideas to address them.

Such information could be used to revise and incorporate key current topics into outdated standard reported outcome forms, such as patient quality of life assessments or surgical outcome forms, while informing public health education and promotion campaigns with themes generated from extant online conversations closer to real time when users experience and report them.

### Conclusions

Our analysis demonstrates that LLMs can effectively and efficiently process some large social media–based health-related data sets and extract themes comparable to human researchers. Although LLMs may not yet match human accuracy, this evolving field holds promise for greatly lowering the time and cost of analyses.

## Appendix

Multimedia Appendix 1 Examples of Claude 1 inductive thematic analysis and Claude 2 inductive thematic analysis.

## Abbreviations

BTM: bi-term topic model
LDA: latent Dirichlet allocation
LLM: large language model
NLP: natural language processing

## Data Availability

Deidentified data that contain associated Twitter IDs associated with the original study, including the original subsets of 193 and 768 posts used in this study, are available in a GitHub repository [48].
