# Supplementary material for: Large Language Models Can Enable Inductive Thematic Analysis of a Social Media Corpus in a Single Prompt: Human Validation Study
Source: JMIR Infodemiology. 2024 Aug 29;4:e59641. doi: 10.2196/59641 (PMC11393503; doi:10.2196/59641)
Supplement: Multimedia Appendix 1 [file infodemiology_v4i1e59641_app1.docx]

**Multimedia Appendix 1**

| **Textbox S1. Example of Claude 1 inductive thematic analysis.** The prompt asked the LLM to read 768 posts and define themes, including with a title, description and post examples. Output from Claude 1 test run 1 is shown. | |
| --- | --- |
| **Theme1**  a) Title: Vaccine skepticism during the COVID-19 pandemic  b) Definition: Over the past year as the COVID-19 pandemic has unfolded, skepticism and distrust of potential coronavirus vaccines has emerged among some groups. Several prominent politicians, including [Politician 3] and [Politician 1], have expressed concerns about trusting claims made by the [Politician 4] administration regarding vaccine development timelines and safety. Meanwhile, vocal anti-vaccine individuals and groups have attempted to leverage fears around COVID-19 to expand their audiences and messages opposing vaccination. Some have alleged links between vaccines and the virus that have no scientific basis. This emerging vaccine skepticism threatens to undermine public health efforts to control the pandemic if and when a safe and effective vaccine becomes available. Example 1: "*Anti-Vaxxers? [Politician 3] and [Politician 2] Are Already Sowi...*" (Post 452) - This headline from a conservative journal alleges that [Politician 3] and [Politician 2] are fueling vaccine skepticism.; Example 2: "*Anti-lockdown and anti-vaxxer protesters have merged....* (Post 654) - This NBC opinion piece discusses how anti-vaccine individuals have capitalized on fears around COVID-19 lockdowns to expand their audiences and messages.  **Theme2**  a) Title: Accusations of being "anti-vaxxers" in the political discourse  b) Definition: As skepticism towards potential coronavirus vaccines has emerged, politicians and public figures have accused each other of being "anti-vaxxers" or fueling anti-vaccine sentiment. This includes President [Politician 4] alleging that [Politician 3] and [Politician 2] are anti-vaxxers for expressing concerns about the possible politicization of the FDA approval process. Others have accused Governor [Politician 2] of taking an anti-vaxxer stance for telling New Yorkers to be skeptical of initial vaccine approvals. Such heated rhetoric risks further undermining public trust and confidence during the ongoing pandemic. Example 1: "[Politician 4] *Wildly Accuses [Politician 3] Of Being A Coronavirus 'Anti-Vaxxer*'" (Post 592) - [Politician 4] levied this accusation against [Politician 3].; Example 2: "A*dd [Politician 2] to the list of anti-vaxxer Dems*." (Post 730) - This post accuses [Politician 2] of taking an anti-vaxxer stance.  **Theme3**  a) Title: Negative reactions to celebrity anti-vaccine stances  b) Definition: Several celebrities who have expressed explicit anti-vaccine or coronavirus conspiracy views have received backlash. This includes actress [Actress 2], who played a nurse on TV show "Home and Away" but has questioned vaccine safety, and was dropped from a charitable role. TV character actress [Actress 1], known for playing protective mother Amy Duncan on "Good Luck Charlie," was criticized for sharing 5G coronavirus conspiracy theories and questioning masks. Such celebrity stances threaten to further normalize anti-vaccine views during the current urgent public health crisis Example 1: "*Anti-vaxxer Home and Away actress [Actress 2] is DROPPED as an ambassador...*" (Post 412); Example 2: "j*ust found out amy duncan is antivax and antimasks...good luck charlie*" (Post 741)  **Theme4**  a) Title: Comparisons between anti-lockdown and anti-vaccine movements  b) Definition: Commentators have drawn comparisons between anti-lockdown protesters opposing coronavirus business and movement restrictions, and anti-vaccine advocates. Both movements question the need for and efficacy of public health measures, leveraging related fears to expand. Coverage discusses how the two movements have begun converging at some protests. Public health experts warn this could undermine control of the pandemic if a vaccine becomes available, as these aligned movements may help normalize skepticism towards vaccination. Example 1: "*Opinion \| Anti-lockdown and anti-vaxxer protesters have merged...*" (Post 653); Example 2: "*The new antiscience: In...*" (Post 420)  **Theme5**  a) Title: Spread of anti-vaccine messaging during the pandemic  b) Definition: Vocal anti-vaccine individuals and groups have leveraged the attention paid to coronavirus to disseminate long-standing health misinformation to new audiences under the cover of pandemic-related fears. This includes baselessly linking vaccines to COVID-19 and the disease, to encourage ongoing general vaccine hesitancy. Such opportunistic growth during a major public health crisis threatens to normalize anti-vaccine narratives and undermine emergency control of COVID-19 if a vaccine is approved. Example 1: "*Anti-vaxxer groups are growing on Facebook...*" (Post 168); Example 2: "*Antivax finding new life through #COVID19*."(Post 736) | |
| **Textbox S2. Example of Claude 2 inductive thematic analysis.** The prompt asked the LLM to read 768 posts and define themes, including with a title, description and post examples. Output from Claude 2 test run 1 is shown. |  |
| Here are 5 significant themes from the provided content, with examples:  **Theme1**  a) Title: Skepticism towards COVID-19 vaccines  b) Definition: Many posts express distrust or skepticism towards potential COVID-19 vaccines, often labeling political figures or groups as "anti-vaxxers" for casting doubt on vaccines. This skepticism stems from factors like the rapid vaccine development timeline and political disputes.  c) Examples: "*Anti-Vaxxers? [Politician 3] and [Politician 2] Are Already Sowing Seeds of Distrust of a COVID-19 Vaccine*"; "*[Politician 4] calls Dems anti-vaxxers, says their anti-vaccine rhetoric is 'dangerous' after Sen. [Politician 2] said she wouldn't trust the him on safety of COVID vaccine before the election*"  **Theme2**  a) Title: Blaming deaths and outbreaks on anti-vaccine views  b) Definition: Some posts blame COVID-19 infections and deaths on anti-vaccine views, suggesting that being against vaccines leads to worse health outcomes.  c) Examples: *"surely not anti-vaxxer [Tennis Pro] testing postive for covid-19 after he organised a tennis tournament complete with fans present and minimum social distancing efforts?"; "Anti-vaxxer tennis star [Tennis Pro] tests POSITIVE for coronavirus after world No 1 and fellow players ignored social distancing by partying and playing football and basketball on his Adria Tour"*   **Theme3**  a) Title: [Tennis Pro]'s COVID-19 diagnosis  b) Definition: Many posts discuss tennis player [Tennis Pro] testing positive for COVID-19 and connect it to his anti-vaccine views, criticizing his decisions.  c) Examples: *"Anti-vaxxer tennis star [Tennis Pro] tests POSITIVE for coronavirus after world No 1 and fellow players ignored social distancing by partying and playing football and basketball on his Adria Tour"; "So not only did noted anti-vaxxer & COVID-19 disbeliever [Tennis Pro] catch COVID-19 during the Adria tennis tournament which he organised in Serbia (w/ zero social distancing amid the pandemic); it was to raise funds for COVID-19 relief. Karma sure is..*"  **Theme4**  a) Title: Insults and criticisms of anti-vaccine people  b) Definition: Some posts directly insult or criticize people deemed as holding anti-vaccine views, ascribing negative attributes to them.  c) Examples: *"[Tennis Pro] ‚Äî anti-vaxxer and pseudoscience promoter ‚Äî is an awful advocate for the sport of tennis."; "This unbelievably idiotic sentiment radiates with anti-vaxxer energy."*  **Theme5**  a) Title: Anti-vaccine views linked to other conspiracies  b) Definition: Several posts connect anti-vaccine views to belief in other conspiracies like 5G networks causing COVID-19. This portrays anti-vaccine views as part of a broader set of unsubstantiated beliefs.  c) Examples: *"So [Tennis Pro] is an anti-vaxxer and his missus is spreading 5G/Corona conspiracies"; " [Tennis Pro] is an anti-vaxxer, [Tennis Pro 3] is a 5g conspiracist. I see 4chan is big in the [Tennis Pro] household."* |  |
